# Deep neural-network based optimization for the design of a multi-element surface magnet for MRI applications

**DOI:** 10.1088/1361-6420/ac492a

**Published:** 2022-01-26

**Authors:** Sumit Tewari, Sahar Yousefi, Andrew Webb

**Affiliations:** 1 C.J. Gorter Center for High Field MRI, Radiology, Leiden University Medical Center, Leiden, The Netherlands; 2 Division of Image Processing, Leiden University Medical Center, Leiden, The Netherlands

**Keywords:** optimization problem, inverse problem, deep neural network, self-training, magnet design, surface-magnets, MRI

## Abstract

We present a combination of a CNN-based encoder with an analytical forward map for solving inverse problems. We call it an encoder-analytic (EA) hybrid model. It does not require a dedicated training dataset and can train itself from the connected forward map in a direct learning fashion. A separate regularization term is not required either, since the forward map also acts as a regularizer. As it is not a generalization model it does not suffer from overfitting. We further show that the model can be customized to either find a specific target solution or one that follows a given heuristic. As an example, we apply this approach to the design of a multi-element surface magnet for low-field magnetic resonance imaging (MRI). We further show that the EA model can outperform the benchmark genetic algorithm model currently used for magnet design in MRI, obtaining almost 10 times better results.

## 1 Introduction

Cause and effect are central to every physical measurement in the classical world. For systems that are described by an exact mathematical model, the parameters of the model represent the cause, and the output of the model the effect. Given a mathematical model, this mapping from a set of input parameters to an output state is straightforward. However, an inverse mapping from the output of such models to a complete set of input parameters, termed an inverse problem, is much more challenging [1]. If the forward map, *ℱ* (from cause to effect) is linear with respect to the model parameters i.e. *y = ℱx*, with *x* being the model parameters, then the inverse problem of finding *x* given *y* and *ℱ* is also linear. However, a non-linear forward map leads to non-linear inverse problems [2], the majority of which are also ill-posed. Linear inverse problems with a finite number of model parameters can be formulated as a linear system of equations with the forward map (*ℱ*) written in the form of a matrix. In the case when *ℱ* is a full rank square matrix, then a unique inverse solution can be obtained by constructing a *ℱ*^−1^ matrix, although it may quickly become intractable for large matrices. If this is not the case, but the linear inverse problem is of the type which can be written using Fredholm’s integral equation of the first kind [3], then this integral equation can be discretized and written in *Ax = b* linear form, where *A* is a matrix that depends on the forward map, *b* is the measured signal output and *x* is the set of model parameters. Non-linear inverse problems are usually more challenging and have no general method for solving them. The most common method is to try to approximate the non-linear forward map to a linear one. A conventional neural network creates a map between input and output by learning from a training data set. Numerical inversion of the forward neural network can be derived, for example, from a back-propagation algorithm [5, 6] assuming duality of the weights. However, this technique can be affected by overfitting unless a very large training dataset is available and small batch sizes are used [44].

For inverse problems which can be formulated into the form of Fredholm integral equation and digitized, a Hopfield neural network has been utilized to solve the problem [7]. The network energy of the Hopfield network decreases with network operation and so the total variation error for the inverse problem can be parameterized to the Hopfield network energy. However, this suffers from the problem that an optimum solution can not be obtained unless a suitable initial state has been set [8, 9]. These techniques all require training of the neural network in the forward direction and are dual port networks (input and output). In contrast, Ogawa *et al* [10] have suggested a triple port network with weights being the third one. The inverse solution is thus extracted from this third port. This technique was termed the answer-in-weights technique. However, up to now this technique has been demonstrated only for smaller networks with a handful of neurons. Recently, multi-layer perceptron artificial neural networks were also used in solving inverse problems for designing photonic systems [11, 12]. Ardiz-zone *et al* [13] have devised an invertible network which incorporates bidirectional training to obtain automatically the inverse map once the network is trained in the forward direction. However, the invertible network has currently only been demonstrated for low-dimensional inputs and outputs. The emergence of deep convolutional neural networks (CNNs) has boosted the impact of artificial intelligence. CNNs help in preserving the dimensionality of the input and thus incorporate intelligent feature extraction. A CNN based encoder–decoder architecture has been used widely for solving inverse problems in imaging, see Lucas *et al* [47] and the citations therein. The basic idea behind this is that the feature maps are spatially compressed by an encoder network, then increased back to the size of the output image by a decoder network. In medical imaging some applications of this are in image denoising, deconvolution, superresolution, image reconstruction [14, 16, 19, 46] and image-to-image compression [36, 39, 40]. A large training data-set for these problems can easily be obtained by adding deformations and noise to openly available online image datasets. A bottleneck in these methods is that the knowledge of neural networks is constrained to the data seen during training. Attempts have been made to combining domain-based knowledge with the deep-learning models [17, 18] to improve their performance.

The inverse problem solutions provided in the literature are mostly case-dependent [20]. So far, no general framework for solving inverse problems using neural networks has been developed. Solving a general inverse problem (either linear or non-linear) related to physical measurements using deep neutral network faces the following challenges. First, many of these inverse problems are regression problems. This means that the output of the model are quantitative numbers and could demand a certain level of accuracy. Second, the training datasest for many such problems are not easily available. The problem becomes especially complicated if trying to train an AI model to predict a solution which is not present in the statistical distribution of the training dataset. An example of this is finding an input corresponding to a target output which is better, with respect to a defined metric, than all other outputs present in the training dataset. Third, solving these inverse problem still requires a prior knowledge of the regularization constraint to be entered into the system [21].

In this present work, in order to overcome some of the limitations outlined above, we suggest a simple method for solving inverse problems using a deep neural network when the analytical expression or the forward map (*ℱ*) is known. This is accomplished by constructing an encoder-analytic (EA) hybrid model. We demonstrate here the capability of such an hybrid model by solving a magnet design problem. We compare our result with the very commonly used genetic algorithm (GA) technique.

## 2 Encoder-analytic hybrid AI model

**Figure 1:**
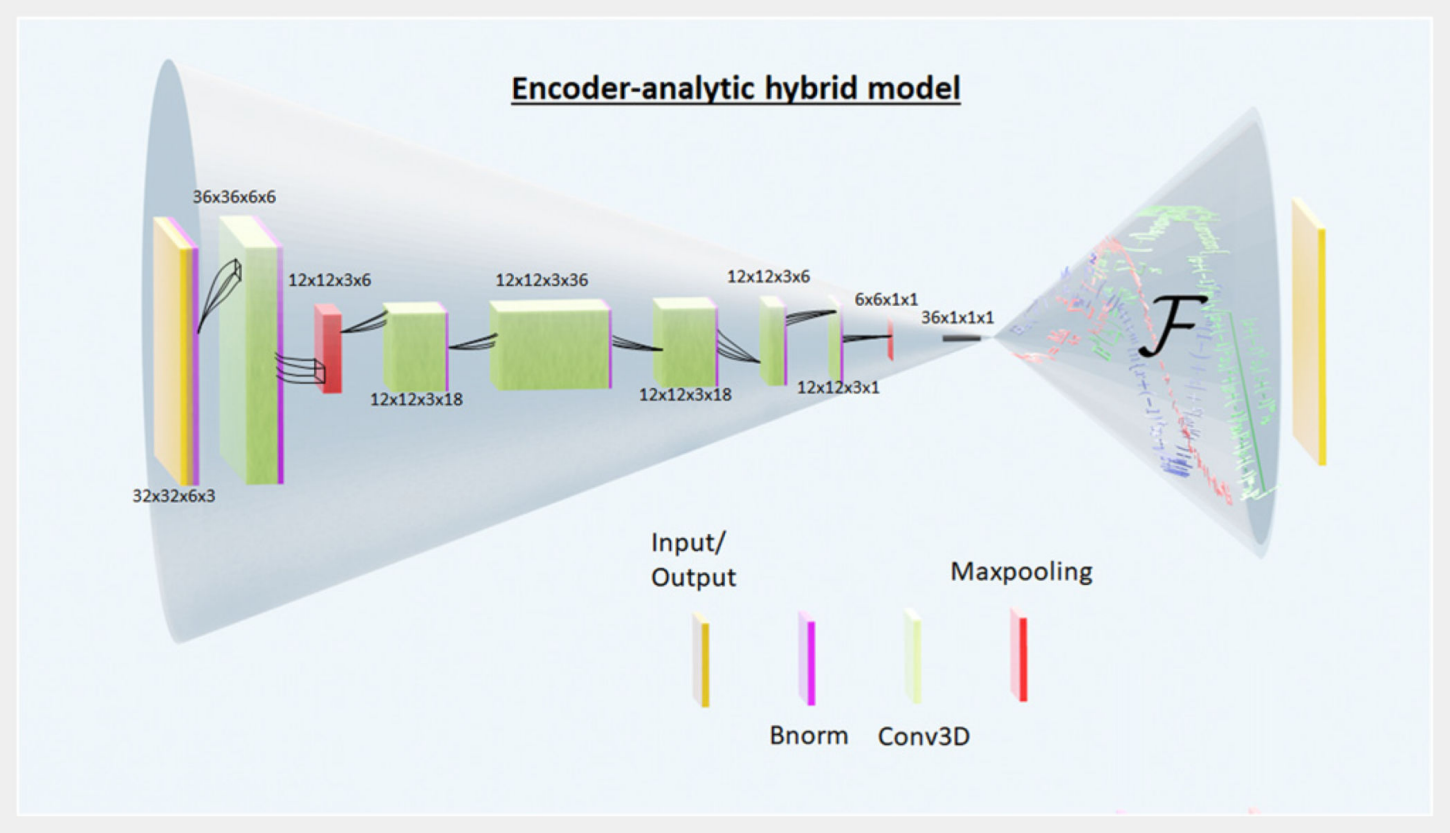
EA hybrid model. The left side of the model is the encoder which takes a 3D target vector field as the input, and outputs a 1D position vector of length 36 for the 36 permanent magnets used here (6 × 6 magnet array, see figure 2). The right side of the model is the analytic part (*ℱ*) and does not have any neural network inside. It takes the magnet design output of the encoder and generates a 3D vector field, which is compared with the target field and the error is propagated backwards.

Our model is a modification of a standard encoder-decoder model. An example case model is shown in figure 1 in which the neural network in the decoder part has been replaced by an analytic equation solver. The analytic equation is simply the known forward map *ℱ*, and the encoder part is a deep neural network based architecture which will be optimized to map the inverse solution using the forward problem defined in the decoder. For our specific example shown in figure 1 the encoder is CNN based model which encodes the information stored in the bigger size input to a lower-resolution output. We term this combination to be an encoder-analytic hybrid model (EA model). The addition of the analytical part avoids introduction of the usual regularization term. Regularization terms provide additional (known) constraints which could be useful in defining complex forward map. If such regularization constraints are required, then they will be part of the forward map definition only in our EA model.

The EA model presented here is an optimization model and not used for generalization, which will become clearer with the example case discussed in the later sections. Thus it utilizes a direct-learning approach, as opposed to indirect-learning in which a large training dataset is created using the analytical forward map. In a regular encoder–decoder AI model, this training dataset is then used to train both the encoder and decoder parts. The accuracy of the optimum solution to the inverse problem, i.e. the output from the encoder, in such an indirect learning method strongly depends on how well the decoder part (the forward map) is trained and how appropriate is the training dataset. In the EA model proposed here, the accuracy is not compromised as there is no neural network to be trained in the decoder part (alternatively this would be analogous to a usual encoder-decoder model where the decoder is trained to 100% accuracy). The optimization EA model presented here does not suffer from overfitting. Overfitting happens when a model becomes too attuned to the data on which it was trained. That means the model could explain the training data very well, but it will fail on another unknown dataset.

In fact, one can see the overfitting of an generalization AI model in the response of a validation or test dataset. In our case, there is no unknown test/validation dataset. The EA model works on one problem at a time and attuning to that problem is not considered as overfitting. Once the problem is defined it will adjust its weights and biases to find the solution for that specific problem and is not supposed to work in a generalized manner by design.

## 3 Magnet array design

The particular example we have chosen to demonstrate the performance of the EA model is that of producing a desired three-dimensional magnetic field distribution using an array of individual rare-earth permanent magnets. Applications of different field distributions include magnetic resonance imaging [22, 27], particle accelerators and stellarators [23, 24] used for plasma confinement.

For a single isolated magnet with magnetization $\vec{M}\left({\vec{r}}_{i}\right)$, the forward map given in equation (1) provides the expression for the magnetic field as a volume (*V*) integral: $\vec{H}(\vec{r})$.

(1) $$\vec{H}(\vec{r})={\vec{\nabla }}_{r}\cdot \frac{1}{4\pi }\underset{V}{\int }\frac{{\vec{\nabla }}_{r_{i}}\cdot \vec{M}\left({\vec{r}}_{i}\right)}{\left|\vec{r}-{\vec{r}}_{i}\right|}{\text{d}}^{3}{\vec{r}}_{i}.$$

$\vec{M}$ is the magnetization vector. Engel-Herbert and Hesjedal [26] have derived an analytical solution to equation (1) for cuboid magnets, represented by equations (5)–(7) in that publication. For arrays of individual magnets, the contributions from each of the elements is added together.

Surface magnets are mostly designed by manually (brute force technique) tuning the position and orientation of a small number of cubic or cuboid permanent magnets [28–31]. Under certain assumptions, such as the desired region of interest (ROI) is far from the magnet surfaces and the magnetization fills a single region with smooth curved boundaries, the inverse of the magnet design problem could be made linear. Such linear-inverse problem could then be solved using Tikhonov type regularization as discussed earlier [4, 24]. However, in situations where the ROI cannot be assumed far from the magnet, the sparsity of the magnets and the non-unity permeability interactions between individual magnets cannot be ignored, and the inverse magnet design problem becomes highly non-linear.

There are two broad classes of optimization target fields: one which consists of a linear static magnetic field gradient perpendicular to the surface of the magnet [28, 32, 33, 35, 41, 45,49] or a localized ‘sweet spot’ consisting of a ROI in which the magnetic field is relatively homogeneous in all three dimensions [29, 30, 34, 42, 43, 48, 50]. In the next section we will use the EA model we developed to find an optimized magnet design solution by varying the relative positions of the individual permanent cubic magnets. The optimized design should thus have a relatively low strength linear gradient perpendicular to the magnet surface which can be used for spatial encoding to the surface of the magnet and at the same time a uniform field in the *x*–*y* plane in a ROI defined at a set distance above the surface of the magnet array.

## 4 Method

**Figure 2:**
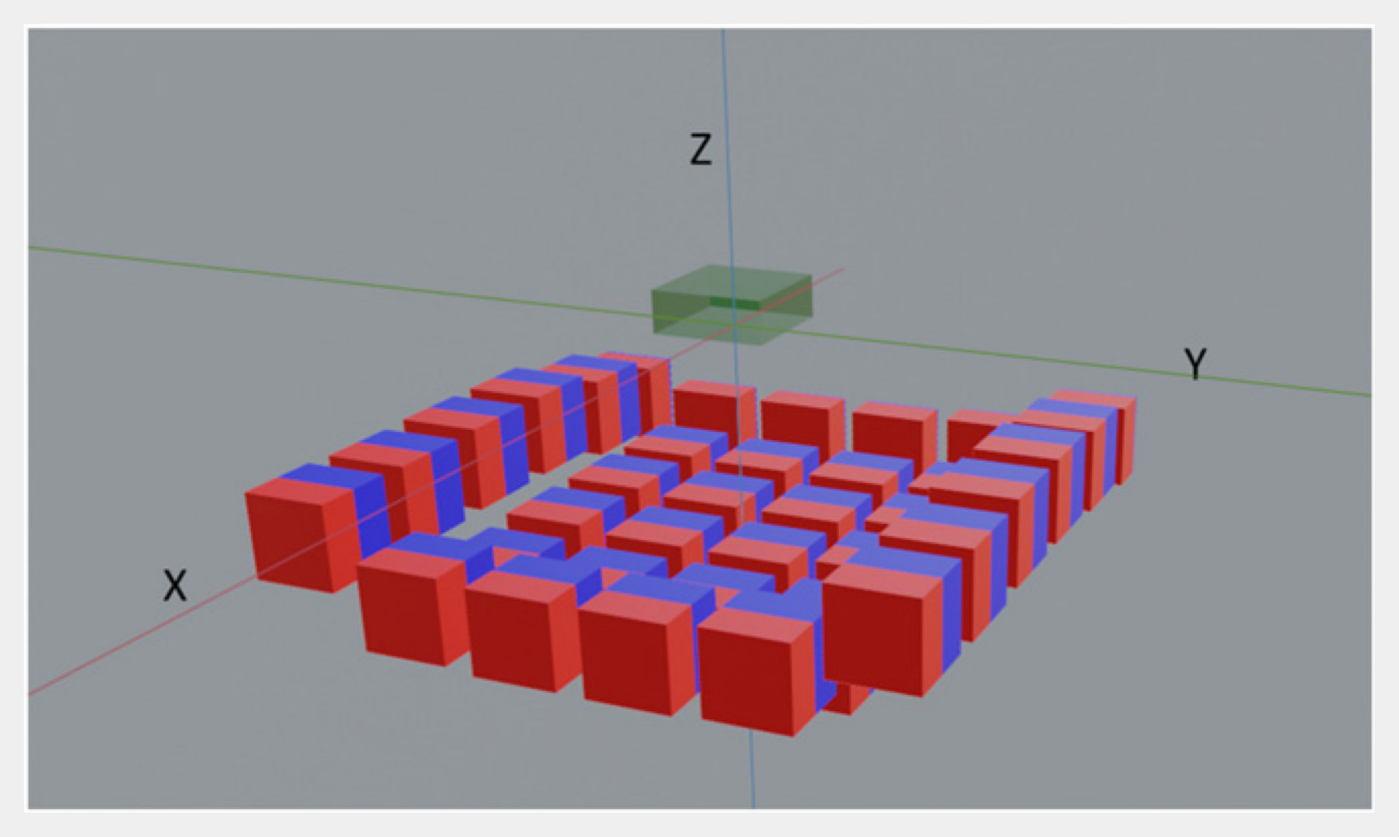
Surface magnet constructed using 36 permanent cubic magnets. The blue and red color corresponds to the north and south pole respectively. The ROI of dimension (16 × 16 × 6 mm^3^) is depicted in green color.

We use a 6 × 6 grid of permanent cubic magnets with the remanent magnetization of 1.32T, that corresponds to widely available NdFeB magnets. The optimization task is to find the best *z*-positions for each magnet for the magnetic field distribution defined above. For simplicity, we keep the orientation of all the magnets same. The limits of the search space for the program for each magnet is ±5 mm translation along the *z*-axis (see figure 2). Note that a brute force technique finding the best solution with 1 mm steps would require scanning 11^36^ permutations. The analytic part in our EA model, the forward map *ℱ*, uses the analytic expression given by Engel-Herbert and Hesjedal [26]. The encoder part shown in figure 1 corresponds to this surface magnet example only. It is based on CNN architecture and is implemented in TensorFlow which is an open-source machine learning software. It is similar to the encoder of an image-to-image compression model [15, 36, 39, 40], which encodes the information stored in the bigger size input to a lower-resolution output. There are a few subtle differences however there. First, the encoder part of regular image-to-image compression model would have the number of channels increasing while decreasing the resolution. While in our case the input of the encoder is a 3D target field image of dimensions 32 × 32 × 6 (*N_x_*_–pixel_ × *N_y_*_–pixel_ × *N_z_*_–pixel_) with three channels (which corresponds to the three components of the vector field) and a convolution neural network is then utilized to compress it to a single channel 6 × 6 grid output (compressed 2D image) which are the positions of a 6 × 6 grid of magnets. Here, *N_x_*_–pixel_, *N_y_*_–pixel_ and *N_z_*_–pixel_ are the the number of pixels in the *x*, *y* and *z*-directions in the ROI respectively. By doing this, we preserve the spatial information of the magnets and connect it to the spatial variation of the magnetic field. Second difference is that in a regular image-to-image compression model the encoder is always trained along-side the decoder part, while in our case as the decoder part is not a neural network so just the encoder gets trained.

One of the standard figures of merit to design the 3D magnetic field from an MRI magnet is (to minimize) the inhomogeneity of the field, which is calculated as $\frac{\text{max}(B)-\text{min}(B)}{\text{mean}(B)}$, where *B* is the dominant component, i.e. *B_x_, B_y_* or *B_z_*, of the field. The magnetic field generated by surface magnets decreases rapidly as a function of distance from the surface (in the *z*-direction). (*G_z_*). To find the design with a linear-gradient the figure of merit that is minimized here is thus defined over a reduced field *B_r_ = B – zG_z_*. This criterion also results in the most homogeneous field in the *xy*-plane. For obtaining a reasonable signal-to-noise ratio, the criterion that only magnetic field values above 24.46 mT, corresponding to a 1 MHz Larmor frequency, were considered. For the current case, a custom loss (error) term is defined which is nothing but these inhomogeneity values. The error is then propagated backwards to update the weights and biases of the encoder model. The model is sent back and forth till a desired figure of merit is achieved. This EA model thus does not require a dedicated training data set. It determines the solution by iteratively learning from the forward map of the problem (the analytic part) and fine-tuning the parameters for subsequently constructing the inverse map (the encoder part). Along with the custom loss function, for optimization we have used the Adam optimizer available in TensorFlow. It is a stochastic gradient descent optimizer that is based on adaptive estimation of first-order and second-order moments. The output of the encoder part is passed through a tanh activation function which generates output between −1 and +1. After proper scaling this is converted to the *z*-position in a desired range. Although we do train the encoder part of the model with the analytic part, but as we do not require any dedicated training dataset, so the batch size is set to one. This could also help to avoid falling into local minima [44].

## 5 Results/discussion

**Figure 3:**
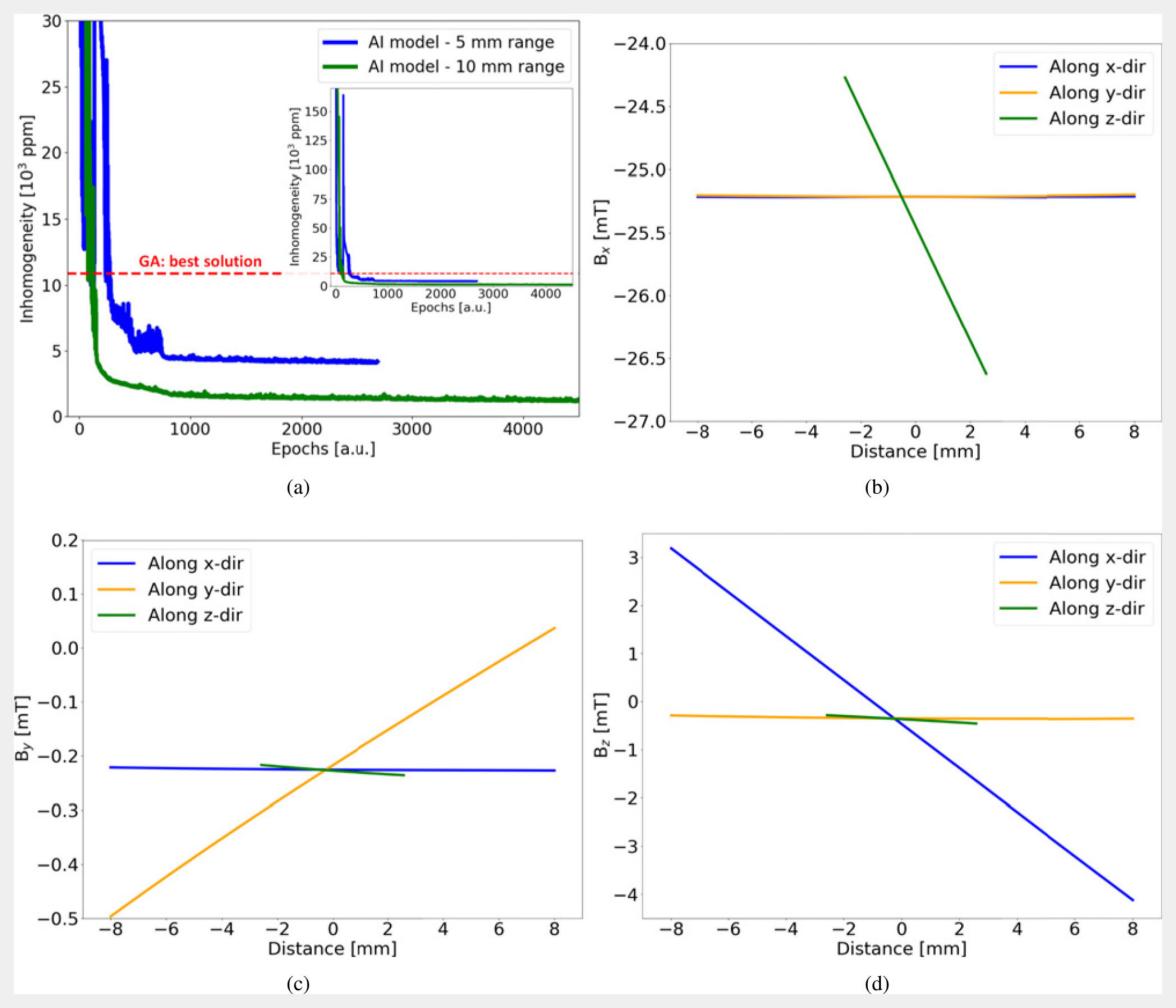
(a) Convergence of the inhomogeneity values in our EA model. The blue and green curves show results from single EA model runs for ±5 mm and ±10 mm search range respectively. The red dashed line marks the best inhomogeneity value obtained using GA model. (b)–(d) Simulation results for the best magnet: *B_x_, B_y_, B_z_* line plots at the center of the ROI along *x-, y-* and *z*-direction. Magnet design data is provided in the supplementary information.

A ROI of dimensions 16 mm × 16 mm × 6 mm was defined centred at a distance 26 mm above the zero position of the magnets. Figure 3(a) shows the result of the inhomogeneity convergence obtained using the EA model for ±5 mm search range. The starting point was all of the magnets with the same *z*-location, which gave an inhomogeneity above 150000 ppm, which rapidly decreased to 5000 ppm after 200 epochs. These results were compared to those from a widely-used GA. The best GA design was with inhomogeneity of 10 889 ppm while the best EA design has 4065 ppm for ±5 mm search range. This shows that the inhomogeneity in the EA model is about 2.5 times better than that obtained using multiple-runs of the GA. An existing python evolutionary algorithm framework called DEAP is used for implementing the GA code and been optimized to design low field MRI systems earlier [37, 38]. Each run of the GA code had 150 generations each with crossover and mutation happening between 10 000 populations in each generations. The minimum inhomogeneity obtained using GA is marked with a red dashed line in figure 3(a).

One possible explanation for the better magnet design output from EA model as compared to the GA model could be found in the finite number of pre-defined constituents that are used to construct the parent states used in GA model. For example, here as the search range was ±5 mm with step of 1 mm that amounts to 11 pre-defined constituents. However, the EA model looks for optimized solution anywhere in the ±5 mm range. Another benefit of EA over GA model is that in the EA model the size of the search space can be increased without effectively increasing the time required to find the solution. This can be seen in figure 3(a), when the search space was increase to ±10 mm, the over all convergence rate was similar while the best results obtained using EA model was brought down to approximately 1122 ppm, which is almost 10 times better than GA output. The corresponding magnet design is given in table 1 in the supplementary information (https://stacks.iop.org/IP/38/035003/mmedia). Here the 6 mm thick ROI is centered at *z =* 0 mm. The time the EA model takes for 10k iterations is around 6 h. Figures 3(b)–(d) show the line plots for the *B_x_,B_y_* and *B_z_* components in the ROI along the *x-, y-* and *z*-directions for the best solution found by the EA model.

We have also investigated the use of the encoder–decoder model proposed by Beliy *et al* [51] and also a simple encoder model where only the inverse map was trained using a training dataset generated using GA. Our results showed that the best magnet design in these cases is very similar to that obtained obtain via the GA model. These models are also very sensitive to how realistic is the target field. On the other hand, the EA model can look for a field which satisfies a certain heuristic and this heuristic might or might not include the target field. Thus the EA model can be used in two modes: either to find a target input or to find a solution that follows a certain heuristic. In the second case, where a pre-defined heuristic will be used as a custom loss, it is not important what the target field looks like. As the EA model gets trained in a direct learning fashion (without any dedicated training data set) to find a solution for a given target field and/or a given heuristic so it is not affected by over-fitting.

## 6 Conclusion

We have shown a hybrid artificial intelligence optimization model to solve inverse problems where the mathematical formulation for the forward map is known. Our hybrid model has two parts similar to encoder–decoder models with the decoder part being replaced by the known analytical forward map. The encoder part is CNN based deep neural network which compresses the input magnetic field data to a 2D magnet design output. We show that such an EA hybrid model can be used to find an optimized inverse solution and does not require any training dataset, since the encoder can learn directly from the analytic part. Normally, solving such inverse problems would require some form of regularization; however in this case the analytic part also acts as a regularizer.

We have demonstrated that we can use such an EA model to design a surface MRI magnet. We compared the results obtained using the EA model with commonly used GA model which are non-deterministic, stochastic in nature. The EA model performs almost 10 times better than a standard GA model. We also note that, in addition to enabling optimal design, an inverse problem solution could also be used for detection of faults [25] in these magnetic system which could be performed by studying the distribution of magnetization.

## Data Availability

All data that support the findings of this study are included within the article (and any supplementary files).
